# Supplementary material for: Distribution Pattern of Mangrove Fish Communities in China
Source: Biology (Basel). 2022 Nov 24;11(12):1696. doi: 10.3390/biology11121696 (PMC9774577; doi:10.3390/biology11121696)
Supplement: Supplementary file 1 [file biology-11-01696-s001.zip › biology-2023115-supplementary.pdf]

Table S1: The checklist of mangrove fishes in China.

No.	Class, Order, Family, Genus, Species	IUCN	Feeding habits	Habitat
	Chondrichthyes			
	Carcharhiniformes			
	Carcharhinidae			
	Carcharhinus			
1	Carcharhinus hemiodon	CR	Carnivore	MAR; BRA; DEM
2	Carcharhinus sorrah	NT	Carnivore	MAR; BRA; RFA
	Rhizoprionodon			
3	Rhizoprionodon acutus	VU	Carnivore	MAR; FRE; BRA; BEP
	Myliobatiformes			
	Dasyatidae			
	Hemitrygon			
4	Hemitrygon akajei	NT	Carnivore	MAR; RFA
5	Hemitrygon bennettii	NE	Carnivore	MAR; DEM
6	Hemitrygon laevigata	VU	Omnivore	MAR; DEM
	Himantura			
7	Himantura microphthalma	NE	Omnivore	MAR; DEM
	Telatrygon			
8	Telatrygon zugei	NT	Carnivore	MAR; BRA; DEM
	Gymnuridae			
	Gymnura			
9	Gymnura bimaculata	NE	Omnivore	MAR; DEM
	Myliobatidae			
	Myliobatis			
10	Myliobatis tobijei	VU	Carnivore	MAR; DEM
	Platyrhinidae			
	Platyrhina			
11	Platyrhina sinensis	EN	Carnivore	MAR; DEM
	Orectolobiformes			
	Hemiscylliidae			
	Chiloscyllium			
12	Chiloscyllium plagiosum	NT	Herbivore	MAR; RFA
	Rajiformes			
	Rajidae			
	Okamejei			
13	Okamejei hollandi	VU	Carnivore	MAR; DEM
	Torpediniformes			
	Narkidae			
	Narke			
14	Narke japonica	VU	Carnivore	MAR; RFA
	Osteichthyes			
	Anguilliformes			
	Anguillidae			
	Anguilla			
15	Anguilla japonica	EN	Carnivore	MAR; FRE; BRA; DEM

No.	Class, Order, Family, Genus, Species	IUCN	Feeding habits	Habitat
	Congridae			
	Ariosoma			
16	Ariosoma anago	DD	Carnivore	MAR; DEM
	Uroconger			
17	Uroconger lepturus	LC	Carnivore	MAR; DEM
	Muraenesocidae			
	Congresox			
18	Congresox talabon	NE	Carnivore	MAR; BRA; DEM; AMP
	Muraenesox			
19	Muraenesox bagio	NE	Carnivore	MAR; BRA; DEM; OD
20	Muraenesox cinereus	LC	Carnivore	MAR; FRE; BRA; DEM; OD
21	Muraenesox yamaguchiensis	NE	Omnivore	MAR; BEP
	Muraenidae			
	Anarchias			
22	Anarchias allardicei	LC	Omnivore	MAR; DEM
	Gymnothorax			
23	Gymnothorax isingteena	LC	Omnivore	MAR; RFA
24	Gymnothorax reevesii	NE	Omnivore	MAR; RFA
25	Gymnothorax reticularis	NE	Carnivore	MAR; DEM
	Strophidon			
26	Strophidon sathete	NE	Carnivore	MAR; FRE; BRA; RFA
	Uropterygius			
27	Uropterygius concolor	LC	Omnivore	MAR; BRA; RFA
	Ophichthidae			
	Lamnostoma			
28	Lamnostoma mindora	NE	Omnivore	FRE; DEM
	Muraenichthys			
29	Muraenichthys gymnopterus	NE	Omnivore	MAR; BRA; RFA
30	Muraenichthys thompsoni	NE	Omnivore	MAR; BRA; DEM
	Myrichthys			
31	Myrichthys maculosus	NE	Carnivore	MAR; RFA
	Ophichthus			
32	Ophichthus apicalis	NE	Omnivore	MAR; BRA; DEM
33	Ophichthus asakusae	NE	Omnivore	MAR; DEM
34	Ophichthus brevicaudatus	NE	Omnivore	MAR; DEM
35	Ophichthus celebicus	NE	Omnivore	MAR; DEM
36	Ophichthus macrochir	NE	Omnivore	MAR; BRA; DEM
	Pisodonophis			
37	Pisodonophis boro	LC	Carnivore	MAR; FRE; BRA; DEM; ANA
38	Pisodonophis cancrivorus	NE	Carnivore	MAR; FRE; BRA; RFA; ANA
	Scolecenchelys			
39	Scolecenchelys macroptera	NE	Omnivore	MAR; RFA
	Synaphobranchidae			
	Dysomma			
40	Dysomma dolichosomatum	NE	Omnivore	MAR; DEM
	Atheriniformes			

No.	Class, Order, Family, Genus, Species	IUCN	Feeding habits	Habitat
	Atherinidae			
	Atherinomorus			
41	Atherinomorus lacunosus	NE	Carnivore	MAR; FRE; BRA; RFA
	Hypoatherina			
42	Hypoatherina tsurugae	NE	Carnivore	MAR; PE
43	Hypoatherina valenciennei	NE	Carnivore	MAR; BRA; PE
	Aulopiformes			
	Paralepidae			
	Lestidium			
44	Lestidium atlanticum	LC	Omnivore	MAR; BAP
	Synodontidae			
	Harpadon			
45	Harpadon nehereus	NT	Carnivore	MAR; BRA; BEP; OD
	Saurida			
46	Saurida elongata	LC	Carnivore	MAR; DEM
47	Saurida tumbil	LC	Carnivore	MAR; RFA; AMP
48	Saurida undosquamis	LC	Carnivore	MAR; RFA; AMP
49	Saurida wanieso	DD	Carnivore	MAR; DEM
	Synodus			
50	Synodus macrops	LC	Omnivore	MAR; DEM
51	Synodus variegatus	LC	Carnivore	MAR; RFA
	Trachinocephalus			
52	Trachinocephalus myops	LC	Carnivore	MAR; RFA
	Beloniformes			
	Adrianichthyidae			
	Oryzias			
53	Oryzias curvinotus	DD	Omnivore	FRE; BRA
	Belonidae			
	Strongylura			
54	Strongylura leiura	NE	Carnivore	MAR; BRA; RFA
55	Strongylura strongylura	NE	Carnivore	MAR; BRA; PE
	Tylosurus			
56	Tylosurus acus	LC	Carnivore	MAR; RFA
57	Tylosurus crocodilus	LC	Carnivore	MAR; RFA
	Exocoetidae			
	Cheilopogon			
58	Cheilopogon spilopterus	NE	Carnivore	MAR; PE
	Hirundichthys			
59	Hirundichthys rondeletii	LC	Omnivore	MAR; PELO
	Hemiramphidae			
	Hemiramphus			
60	Hemiramphus lutkei	NE	Omnivore	MAR; BRA; RFA
	Hyporhamphus			
61	Hyporhamphus affinis	NE	Carnivore	MAR; RFA
62	Hyporhamphus dussumieri	NE	Carnivore	MAR; RFA
63	Hyporhamphus gernaerti	NE	Omnivore	MAR; PE
64	Hyporhamphus intermedius	NE	Carnivore	MAR; FRE; BRA; PE; AMP

No.	Class, Order, Family, Genus, Species	IUCN	Feeding habits	Habitat
65	Hyporhamphus limbatus	LC	Carnivore	MAR; FRE; BRA; PE
66	Hyporhamphus quoyi	NE	Carnivore	MAR; FRE; BRA; PE
	Zenarchopterus			
67	Zenarchopterus buffonis	LC	Herbivore	MAR; BRA; RFA
68	Zenarchopterus philippinus	LC	Omnivore	FRE; PEL
69	Zenarchopterus striga	LC	Omnivore	FRE; BRA; PEL
	Cichliformes			
	Cichlidae			
	Coptodon			
70	Coptodon zillii	LC	Omnivore	FRE; BRA
	Oreochromis			
71	Oreochromis aureus*	NE	Omnivore	FRE; BRA; BEP
72	Oreochromis hybrid	NE	Omnivore	FRE; BRA; BEP
73	Oreochromis mossambicus*	VU	Omnivore	FRE; BRA; BEP
74	Oreochromis niloticus*	LC	Omnivore	FRE; BRA; BEP
	Clupeiformes			
	Chirocentridae			
	Chirocentrus			
75	Chirocentrus dorab	LC	Carnivore	MAR; BRA; RFA
	Clupeidae			
	Clupanodon			
76	Clupanodon thrissa	LC	Omnivore	MAR; FRE; BRA; PE; ANA
	Dussumieria			
77	Dussumieria elopsoides	LC	Carnivore	MAR; PE
	Escualosa			
78	Escualosa thoracata	LC	Carnivore	MAR; FRE; BRA; PE; AMP
	Etrumeus			
79	Etrumeus sadina	LC	Carnivore	MAR; PE; OD
	Herklotsichthys			
80	Herklotsichthys ovalis	DD	Omnivore	MAR; PE
81	Herklotsichthys quadrimaculatus	LC	Carnivore	MAR; FRE; BRA; RFA
	Konosirus			
82	Konosirus punctatus	LC	Carnivore	MAR; BRA; PE; OD
	Nematalosa			
83	Nematalosa come	LC	Carnivore	MAR; PE
84	Nematalosa japonica	DD	Detrivore	MAR; BEP
85	Nematalosa nasus	LC	Herbivore	MAR; FRE; BRA; PE
	Sardinella			
86	Sardinella albella	LC	Herbivore	MAR; BRA; RFA
87	Sardinella aurita	LC	Herbivore	MAR; BRA; PE; OD
88	Sardinella brachysoma	LC	Omnivore	MAR; PE
89	Sardinella fimbriata	LC	Herbivore	MAR; BRA; PE
90	Sardinella hualiensis	LC	Omnivore	MAR; PE
91	Sardinella jussieu	DD	Omnivore	MAR; PE
92	Sardinella lemuru	NT	Herbivore	MAR; PE
93	Sardinella melanura	LC	Omnivore	MAR; PE; AMP

No.	Class, Order, Family, Genus, Species	IUCN	Feeding habits	Habitat
94	Sardinella richardsoni	DD	Omnivore	MAR; PE
95	Sardinella sindensis	LC	Omnivore	MAR; PE
96	Sardinella zunasi	LC	Carnivore	MAR; PE; OD
	Spratelloides			
97	Spratelloides gracilis	LC	Carnivore	MAR; PE
	Tenualosa			
98	Tenualosa ilisha	LC	Detrivore	MAR; FRE; BRA; PE
	Engraulidae			
	Coilia			
99	Coilia grayii	LC	Omnivore	MAR; FRE; BRA; PE
100	Coilia mystus	EN	Carnivore	MAR; FRE; BRA; PE
	Encrasicholina			
101	Encrasicholina punctifer	LC	Omnivore	MAR; RFA; OD
	Engraulis			
102	Engraulis japonicus	LC	Herbivore	MAR; PE
	Setipinna			
103	Setipinna taty	LC	Carnivore	MAR; BRA; PE
104	Setipinna tenuifilis	DD	Carnivore	MAR; FRE; BRA; PE
	Stolephorus			
105	Stolephorus chinensis	LC	Omnivore	MAR; PE
106	Stolephorus commersonnii	LC	Carnivore	MAR; BRA; PE
107	Stolephorus indicus	LC	Carnivore	MAR; BRA; PE; OD
108	Stolephorus insularis	DD	Omnivore	MAR; BRA; RFA
109	Stolephorus tri	NE	Omnivore	MAR; BRA; PE
	Thryssa			
110	Thryssa chefuensis	DD	Omnivore	MAR; PE
111	Thryssa dussumieri	LC	Carnivore	MAR; BRA; PE
112	Thryssa kammalensis	DD	Carnivore	MAR; BRA; PE
113	Thryssa mystax	LC	Carnivore	MAR; BRA; PELO
114	Thryssa setirostris	LC	Carnivore	MAR; BRA; PE
115	Thryssa vitrirostris	LC	Omnivore	MAR; BRA; PE
	Pristigasteridae			
	Ilisha			
116	Ilisha elongata	LC	Carnivore	MAR; BRA; PE
117	Ilisha melastoma	LC	Carnivore	MAR; BRA; PE; AMP
	Cypriniformes			
	Cobitidae			
	Misgurnus			
118	Misgurnus anguillicaudatus	LC	Carnivore	FRE; BRA; DEM
	Cyprinidae			
	Barbodes			
119	Barbodes snyderi	NE	Omnivore	FRE
	Carassius			
120	Carassius auratus*	LC	Detrivore	FRE; BRA; BEP
	Chanodichthys			
121	Chanodichthys erythropterus	LC	Herbivore	FRE; BRA
	Cirrhinus			

No.	Class, Order, Family, Genus, Species	IUCN	Feeding habits	Habitat
122	Cirrhinus molitorella	NT	Detrivore	FRE; BEP
	Cyprinus			
123	Cyprinus carpio*	VU	Herbivore	FRE; BRA
	Hemibarbus			
124	Hemibarbus maculatus	NE	Carnivore	FRE; BEP
	Hemiculter			
125	Hemiculter leucisculus	LC	Herbivore	FRE; BRA
	Henicorhynchus			
126	Henicorhynchus siamensis	NE	Detrivore	FRE; BEP
	Hypophthalmichthys			
127	Hypophthalmichthys molitrix	NT	Detrivore	FRE; BRA; BEP
	Megalobrama			
128	Megalobrama terminalis	NE	Omnivore	FRE; BEP
	Metzia			
129	Metzia lineata	LC	Omnivore	FRE
130	Metzia mesembrinum	NE	Omnivore	FRE; PEL
	Pseudorasbora			
131	Pseudorasbora parva	LC	Carnivore	FRE; BRA; BEP
	Squalidus			
132	Squalidus argentatus	DD	Omnivore	FRE; BRA; BEP
	Cyprinodontiformes			
	Poeciliidae			
	Gambusia			
133	Gambusia affinis*	LC	Carnivore	FRE; BRA
	Poecilia			
134	Poecilia reticulata*	LC	Carnivore	FRE; BRA; BEP
135	Poecilia velifera*	VU	Herbivore	FRE; BRA; BEP
	Elopiformes			
	Albulidae			
	Albula			
136	Albula vulpes	NT	Carnivore	MAR; BRA; RFA
	Elopidae			
	Elops			
137	Elops hawaiensis	DD	Carnivore	MAR; FRE; BRA; PE
138	Elops machnata	LC	Carnivore	MAR; BRA; PE
139	Elops saurus	LC	Carnivore	MAR; BRA; RFA
	Megalopidae			
	Megalops			
140	Megalops cyprinoides	DD	Carnivore	MAR; FRE; BRA; BEP; AMP
	Gadiformes			
	Moridae			
	Lotella			
141	Lotella tosaensis	NE	Omnivore	MAR; DEM
	Gasterosteiformes			
	Syngnathidae			
	Corythoichthys			

No.	Class, Order, Family, Genus, Species	IUCN	Feeding habits	Habitat
142	Corythoichthys flavofasciatus	LC	Omnivore	MAR; RFA
	Hippichthys			
143	Hippichthys cyanospilos	LC	Omnivore	MAR; FRE; BRA; DEM
144	Hippichthys heptagonus	LC	Omnivore	FRE; BRA; DEM
145	Hippichthys penicillus	LC	Omnivore	MAR; FRE; BRA; DEM
146	Hippichthys spicifer*	LC	Omnivore	MAR; FRE; BRA; DEM
	Hippocampus			
147	Hippocampus kuda*	VU	Omnivore	MAR; BRA; RFA
	Phoxocampus			
148	Phoxocampus belcheri	LC	Omnivore	MAR; RFA
	Syngnathus			
149	Syngnathus acus	LC	Carnivore	MAR; BRA; DEM
	Gobiesociformes			
	Gobiesocidae			
	Acyrtus			
150	Acyrtus rubiginosus	LC	Carnivore	MAR; DEM
	Gonorhynchiformes			
	Chanidae			
	Chanos			
151	Chanos chanos	LC	Detrivore	MAR; FRE; BRA; BEP; AMP
	Mugiliformes			
	Mugilidae			
	Crenimugil			
152	Crenimugil buchanani	NE	Detrivore	MAR; FRE; BRA; PE
153	Crenimugil seheli	LC	Herbivore	MAR; FRE; BRA; RFA
	Ellochelon			
154	Ellochelon vaigiensis	LC	Herbivore	MAR; FRE; BRA; RFA
	Mugil			
155	Mugil cephalus*	LC	Omnivore	MAR; FRE; BRA; BEP
	Osteomugil			
156	Osteomugil cunnesius	NE	Herbivore	MAR; FRE; BRA; DEM
157	Osteomugil perusii	LC	Omnivore	MAR; BRA; RFA
158	Osteomugil strongylocephalus	NE	Herbivore	
	Paramugil			
159	Paramugil parmatus	LC	Omnivore	MAR; FRE; BRA; DEM
	Planiliza			
160	Planiliza affinis	NE	Detrivore	MAR; BRA; PE
161	Planiliza alata	LC	Herbivore	MAR; FRE; BRA; BEP
162	Planiliza carinata	NE	Omnivore	MAR; BRA; PE
163	Planiliza haematocheilus	NE	Herbivore	MAR; FRE; BRA; PE
164	Planiliza macrolepis	LC	Herbivore	MAR; FRE; BRA; DEM
165	Planiliza melinoptera	NE	Herbivore	MAR; FRE; BRA; RFA
166	Planiliza subviridis	NE	Herbivore	MAR; FRE; BRA; DEM
	Polynemidae			
	Eleutheronema			
167	Eleutheronema tetradactylum	NE	Carnivore	MAR; FRE; BRA; PE; AMP
	Polydactylus			

No.	Class, Order, Family, Genus, Species	IUCN	Feeding habits	Habitat
168	Polydactylus sextarius	NE	Carnivore	MAR; BRA; DEM; AMP
	Sphyraenidae			
	Sphyraena			
169	Sphyraena forsteri	NE	Carnivore	MAR; RFA
170	Sphyraena pinguis	NE	Carnivore	MAR; PE
	Myctophiformes			
	Myctophidae			
	Benthosema			
171	Benthosema pterotum	LC	Carnivore	MAR; BEP
	Pegasiformes			
	Pegasidae			
	Pegasus			
172	Pegasus volitans	DD	Carnivore	MAR; BRA; DEM
	Perciformes			
	Acanthuridae			
	Acanthurus			
173	Acanthurus xanthopterus	LC	Herbivore	MAR; RFA
	Ambassidae			
	Ambassis			
174	Ambassis gymnocephalus	LC	Carnivore	MAR; FRE; BRA; DEM
175	Ambassis interruptus	LC	Herbivore	MAR; FRE; BRA; DEM; AMP
176	Ambassis kopsii	NE	Carnivore	MAR; FRE; BRA; DEM; AMP
177	Ambassis marianus	LC	Omnivore	
178	Ambassis miops	LC	Carnivore	MAR; FRE; BRA; DEM; AMP
179	Ambassis urotaenia	LC	Omnivore	MAR; FRE; BRA; DEM; AMP
	Anabantidae			
	Anabas			
180	Anabas testudineus*	LC	Herbivore	FRE; BRA; DEM
	Apogonidae			
	Apogon			
181	Apogon striatus	NE	Omnivore	MAR; RFA
	Apogonichthyoides			
182	Apogonichthyoides taeniatus	NE	Carnivore	MAR; RFA
	Jaydia			
183	Jaydia ellioti	NE	Carnivore	MAR; RFA
184	Jaydia lineata	NE	Carnivore	MAR; DEM
	Ostorhinchus			
185	Ostorhinchus cookii	NE	Carnivore	MAR; RFA
186	Ostorhinchus fasciatus	NE	Carnivore	
187	Ostorhinchus kiensis	NE	Omnivore	MAR; BRA; RFA
188	Ostorhinchus notatus	NE	Carnivore	MAR; RFA
	Taeniamia			
189	Taeniamia fucata	NE	Carnivore	MAR; RFA
190	Taeniamia lineolata	NE	Carnivore	MAR; RFA
	Blenniidae			
	Istiblennius			

No.	Class, Order, Family, Genus, Species	IUCN	Feeding habits	Habitat
191	Istiblennius dussumieri	LC	Detrivore	MAR; BRA; RFA
	Omobranchus			
192	Omobranchus elegans	LC	Detrivore	MAR; DEM
193	Omobranchus fasciolatoceps	LC	Omnivore	MAR; DEM
194	Omobranchus punctatus	LC	Omnivore	MAR; BRA; BEP
	Petroscirtes			
195	Petroscirtes breviceps	LC	Detrivore	MAR; BRA; RFA
	Praealticus			
196	Praealticus striatus	LC	Detrivore	MAR; DEM
	Salarias			
197	Salarias fasciatus	LC	Detrivore	MAR; BRA; RFA
	Scartella			
198	Scartella cristata	LC	Detrivore	MAR; RFA
	Callionymidae			
	Callionymus			
199	Callionymus beniteguri	NE	Omnivore	MAR; DEM
200	Callionymus curvicornis	NE	Carnivore	MAR; FRE; BRA; DEM
201	Callionymus doryssus	NE	Omnivore	MAR; DEM
202	Callionymus filamentosus	NE	Carnivore	MAR; DEM
203	Callionymus hainanensis	NE	Omnivore	MAR; DEM
204	Callionymus hindsii	NE	Omnivore	MAR; DEM
205	Callionymus marquesensis	NE	Omnivore	MAR; DEM
206	Callionymus planus	NE	Omnivore	MAR; DEM
207	Callionymus sagitta	LC	Herbivore	MAR; BRA; DEM
208	Callionymus schaapii	NE	Omnivore	MAR; BRA; DEM
209	Callionymus valenciennei	NE	Omnivore	MAR; DEM
	Repomucenus			
210	Repomucenus olidus	NE	Omnivore	FRE; BRA; DEM
211	Repomucenus virgis	NE	Omnivore	MAR; DEM
	Carangidae			
	Alectis			
212	Alectis ciliaris	LC	Carnivore	MAR; RFA
213	Alectis indica	LC	Carnivore	MAR; BRA; RFA
	Alepes			
214	Alepes djedaba	LC	Carnivore	MAR; RFA; AMP
215	Alepes kleinii	LC	Carnivore	MAR; RFA
216	Alepes melanoptera	LC	Carnivore	MAR; BRA; PE
217	Alepes vari	LC	Carnivore	MAR; BRA; PE
	Atropus			
218	Atropus atropos	LC	Carnivore	MAR; PE; AMP
	Atule			
219	Atule mate	LC	Carnivore	MAR; BRA; RFA
	Carangoides			
220	Carangoides equula	LC	Carnivore	MAR; RFA
221	Carangoides malabaricus	LC	Carnivore	MAR; RFA; AMP
222	Carangoides orthogrammus	LC	Carnivore	MAR; RFA; OD
223	Carangoides praeustus	LC	Omnivore	MAR; DEM; OD

No.	Class, Order, Family, Genus, Species	IUCN	Feeding habits	Habitat
	Caranx			
224	Caranx ignobilis	LC	Carnivore	MAR; BRA; RFA
225	Caranx melampygus	LC	Carnivore	MAR; BRA; RFA
226	Caranx papuensis	LC	Carnivore	MAR; BRA; RFA
227	Caranx sexfasciatus	LC	Carnivore	MAR; FRE; BRA; RFA; AMP
	Decapterus			
228	Decapterus maruadsi	LC	Carnivore	MAR; RFA
229	Decapterus russelli	LC	Carnivore	MAR; BEP
	Elagatis			
230	Elagatis bipinnulata	LC	Carnivore	MAR; RFA
	Megalaspis			
231	Megalaspis cordyla	LC	Carnivore	MAR; BRA; RFA
	Parastromateus			
232	Parastromateus niger	LC	Carnivore	MAR; BRA; RFA; AMP
	Scomberoides			
233	Scomberoides commersonnianus	LC	Carnivore	MAR; BRA; RFA; AMP
234	Scomberoides lysan	LC	Carnivore	MAR; BRA; RFA
235	Scomberoides tala	LC	Carnivore	MAR; RFA
236	Scomberoides tol	LC	Carnivore	MAR; BRA; RFA
	Selaroides			
237	Selaroides leptolepis	LC	Carnivore	MAR; BRA; RFA; AMP
	Trachinotus			
238	Trachinotus baillonii	LC	Carnivore	MAR; BRA; RFA
239	Trachinotus blochii	LC	Carnivore	MAR; BRA; RFA
240	Trachinotus ovatus	LC	Carnivore	MAR; BRA; PE
	Trachurus			
241	Trachurus japonicus	NT	Carnivore	MAR; PE; OD
	Uraspis			
242	Uraspis uraspis	LC	Carnivore	MAR; RFA
	Centrolophidae			
	Psenopsis			
243	Psenopsis anomala	LC	Carnivore	MAR; BEP
	Cepolidae			
	Acanthocepola			
244	Acanthocepola limbata	NE	Omnivore	MAR; DEM
	Chaetodontidae			
	Chaetodon			
245	Chaetodon auriga	LC	Carnivore	MAR; RFA
246	Chaetodon vagabundus*	LC	Carnivore	MAR; RFA
	Roa			
247	Roa modesta	LC	Carnivore	MAR; RFA
	Channidae			
	Channa			
248	Channa gachua	LC	Carnivore	FRE; BEP
249	Channa maculata	LC	Carnivore	FRE; BEP
	Drepaneidae			

No.	Class, Order, Family, Genus, Species	IUCN	Feeding habits	Habitat
	Drepane			
250	Drepane longimana	NE	Carnivore	MAR; BRA; RFA
251	Drepane punctata	NE	Carnivore	MAR; FRE; BRA; RFA
	Echeneidae			
	Echeneis			
252	Echeneis naucrates	LC	Carnivore	MAR; BRA; RFA
	Eleotridae			
	Bostrychus			
253	Bostrychus sinensis	LC	Carnivore	MAR; FRE; BRA; DEM; AMP
	Butis			
254	Butis butis	LC	Carnivore	MAR; FRE; BRA; DEM
255	Butis gymnopomus	LC	Omnivore	FRE; BRA; DEM
256	Butis koilomatodon	NE	Carnivore	MAR; FRE
257	Butis melanostigma	NE	Omnivore	MAR; FRE; BRA; DEM; AMP
	Eleotris			
258	Eleotris acanthopoma	LC	Carnivore	MAR; FRE; BRA; DEM; AMP
259	Eleotris fusca*	LC	Carnivore	MAR; FRE; BRA; DEM; AMP
260	Eleotris melanosoma	LC	Omnivore	MAR; FRE; BRA; DEM; AMP
261	Eleotris oxycephala	LC	Omnivore	MAR; FRE; BRA; DEM; AMP
	Ophiocara			
262	Ophiocara porocephala	LC	Carnivore	MAR; FRE; BRA; DEM; AMP
	Prionobutis			
263	Prionobutis dasyrhynchus	DD	Omnivore	FRE; DEM
	Ephippidae			
	Ephippus			
264	Ephippus orbis	NE	Carnivore	MAR; RFA
	Platax			
265	Platax orbicularis	LC	Carnivore	MAR; BRA; RFA
	Gerridae			
	Gerres			
266	Gerres erythrourus	LC	Carnivore	MAR; BRA; RFA; OD
267	Gerres filamentosus	LC	Carnivore	MAR; FRE; BRA; DEM; AMP
268	Gerres japonicus	NE	Omnivore	MAR; DEM
269	Gerres limbatus	LC	Carnivore	MAR; BRA; DEM; AMP
270	Gerres longirostris	LC	Carnivore	MAR; FRE; BRA; RFA; AMP
271	Gerres macracanthus	NE	Omnivore	MAR; BRA; DEM
272	Gerres oblongus	LC	Carnivore	MAR; RFA
273	Gerres oyena	LC	Carnivore	MAR; BRA; RFA
	Pentaprion			
274	Pentaprion longimanus	LC	Carnivore	MAR; BRA; DEM
	Gobiidae			
	Acanthogobius			
275	Acanthogobius flavimanus	LC	Carnivore	MAR; FRE; BRA; DEM; AMP
276	Acanthogobius hasta	LC	Carnivore	MAR; FRE; BRA; DEM; AMP
	Acentrogobius			
277	Acentrogobius caninus	LC	Herbivore	MAR; FRE; BRA; DEM; AMP
278	Acentrogobius chlorostigmatoides	NE	Omnivore	FRE; BRA; DEM

No.	Class, Order, Family, Genus, Species	IUCN	Feeding habits	Habitat
279	Acentrogobius janthinopterus	LC	Omnivore	MAR; FRE; BRA; RFA; AMP
280	Acentrogobius nebulosus	LC	Omnivore	MAR; FRE; BRA; RFA; AMP
281	Acentrogobius pflaumii	NE	Carnivore	MAR; BRA; DEM
282	Acentrogobius viganensis	NE	Omnivore	MAR; BRA; DEM
283	Acentrogobius viridipunctatus	LC	Omnivore	MAR; FRE; BRA; DEM; AMP
	Amblychaeturichthys			
284	Amblychaeturichthys hexanema	NE	Carnivore	MAR; DEM
	Amblyeleotris			
285	Amblyeleotris guttata	LC	Omnivore	MAR; RFA
	Amblygobius			
286	Amblygobius albimaculatus	LC	Herbivore	MAR; BRA; RFA
287	Amblygobius phalaena	LC	Herbivore	MAR; RFA
	Amblyotrypauchen			
288	Amblyotrypauchen arctocephalus	LC	Omnivore	MAR; DEM
	Amoya			
289	Amoya microps	NE	Omnivore	
	Apocryptodon			
290	Apocryptodon madurensis	NE	Detrivore	MAR; FRE; BRA; DEM; AMP
291	Apocryptodon punctatus	LC	Omnivore	BRA; DEM
	Arcygobius			
292	Arcygobius baliurus	DD	Omnivore	MAR; BRA; DEM
	Aulopareia			
293	Aulopareia atripinnatus	NE	Omnivore	BRA; DEM
	Awaous			
294	Awaous melanocephalus	NE	Omnivore	MAR; FRE; BRA; BEP; AMP
	Bathygobius			
295	Bathygobius cotticeps	LC	Omnivore	MAR; BRA; RFA
296	Bathygobius cyclopterus	LC	Omnivore	MAR; BRA; RFA
297	Bathygobius fuscus	LC	Carnivore	MAR; FRE; BRA; RFA; AMP
	Caragobius			
298	Caragobius urolepis	LC	Carnivore	FRE; BRA; DEM; AMP
	Chaenogobius			
299	Chaenogobius annularis	LC	Omnivore	FRE; DEM
	Chaeturichthys			
300	Chaeturichthys stigmatias	NE	Carnivore	MAR; DEM
	Cotylopus			
301	Cotylopus rubripinnis	VU	Omnivore	FRE; BEP
	Cryptocentrus			
302	Cryptocentrus pavoninoides	DD	Omnivore	MAR; DEM
303	Cryptocentrus yatsui	NE	Omnivore	MAR; DEM
	Ctenogobiops			
304	Ctenogobiops formosa	NE	Omnivore	MAR; RFA
	Ctenogobius			
305	Ctenogobius brevirostris	NE	Omnivore	
306	Ctenogobius chusanensis	NE	Omnivore	MAR; BRA; DEM
307	Ctenogobius gymnauehen	NE	Omnivore	

No.	Class, Order, Family, Genus, Species	IUCN	Feeding habits	Habitat
	Ctenotrypauchen			
308	Ctenotrypauchen chinensis	NE	Omnivore	MAR; FRE; BRA; DEM
	Exyrias			
309	Exyrias belissimus	LC	Herbivore	MAR; RFA
310	Exyrias puntang	LC	Carnivore	MAR; BRA; RFA
	Favonigobius			
311	Favonigobius gymnauchen	NE	Herbivore	MAR; FRE; BRA; RFA; AMP
312	Favonigobius reichei	LC	Omnivore	MAR; FRE; BRA; DEM; AMP
	Glossogobius			
313	Glossogobius aureus	LC	Carnivore	FRE; BRA; DEM; AMP
314	Glossogobius bicirrhosus	LC	Omnivore	MAR; FRE; BRA; DEM; AMP
315	Glossogobius circumspectus	LC	Omnivore	FRE; BRA; DEM
316	Glossogobius giuris*	LC	Carnivore	MAR; FRE; BRA; BEP; AMP
317	Glossogobius olivaceus	LC	Omnivore	MAR; FRE; BRA; DEM; AMP
	Gnatholepis			
318	Gnatholepis cauerensis	LC	Herbivore	MAR; BRA; RFA
	Gobiopterus			
319	Gobiopterus lacustris	DD	Omnivore	FRE; DEM
	Gobius			
320	Gobius poecilichthys	LC	Omnivore	
	Gymnogobius			
321	Gymnogobius urotaenia	NE	Omnivore	
	Hemigobius			
322	Hemigobius hoevenii	LC	Omnivore	MAR; FRE; BRA; DEM; AMP
	Istigobius			
323	Istigobius campbelli	NE	Omnivore	MAR; DEM
324	Istigobius hoshinonis	LC	Omnivore	MAR; DEM
	Luciogobius			
325	Luciogobius guttatus	NE	Omnivore	MAR; FRE; BRA; DEM; AMP
	Mugilogobius			
326	Mugilogobius abei	LC	Omnivore	MAR; BRA; DEM
327	Mugilogobius chulae	LC	Carnivore	MAR; FRE; BRA; DEM; AMP
328	Mugilogobius hoevenii	NE	Omnivore	
329	Mugilogobius myxodermus	NE	Omnivore	FRE; DEM
	Myersina			
330	Myersina filifer	LC	Omnivore	MAR; DEM
331	Myersina papuanus	NE	Omnivore	MAR; DEM
	Odontamblyopus			
332	Odontamblyopus rubicundus	LC	Omnivore	MAR; BRA; BEP
	Oligolepis			
333	Oligolepis acutipennis	LC	Carnivore	MAR; FRE; BRA; DEM; AMP
334	Oligolepis fasciatus	NE	Carnivore	
335	Oligolepis jaarmani	LC	Omnivore	BRA; DEM
336	Oligolepis moloana	NE	Carnivore	
	Oxudercess			
337	Oxuderces dentatus	DD	Omnivore	FRE; BRA; DEM; AMP
	Oxyurichthys			

No.	Class, Order, Family, Genus, Species	IUCN	Feeding habits	Habitat
338	Oxyurichthys cornutus	NE	Omnivore	MAR; DEM
339	Oxyurichthys microlepis	LC	Omnivore	MAR; BRA; DEM; AMP
340	Oxyurichthys ophthalmonema	LC	Omnivore	MAR; FRE; BRA; DEM
341	Oxyurichthys papuensis	LC	Carnivore	MAR; BRA; RFA
342	Oxyurichthys tentacularis	DD	Carnivore	MAR; BRA; DEM; AMP
	Parachaeturichthys			
343	Parachaeturichthys polynema	LC	Carnivore	MAR; DEM
	Parapocryptes			
344	Parapocryptes serperaster	LC	Omnivore	MAR; BRA; DEM
	Paratrypauchen			
345	Paratrypauchen microcephalus	LC	Omnivore	MAR; BRA; DEM
	Periophthalmus			
346	Periophthalmus argentilineatus	LC	Carnivore	MAR; FRE; BRA; RFA; AMP
347	Periophthalmus magnuspinnatus	NE	Omnivore	FRE; DEM
348	Periophthalmus modestus	NE	Omnivore	MAR; FRE; BRA; DEM; AMP
	Psammogobius			
349	Psammogobius biocellatus	LC	Omnivore	MAR; FRE; BRA; BEP; AMP
	Pseudapocryptes			
350	Pseudapocryptes elongatus	LC	Omnivore	FRE; BRA; DEM; AMP
	Pseudogobius			
351	Pseudogobius javanicus	NE	Carnivore	MAR; FRE; BRA; BEP
352	Pseudogobius masago	NE	Omnivore	BRA; DEM
353	Pseudogobius taijiangensis	NE	Omnivore	BRA; DEM
	Rhinogobius			
354	Rhinogobius davidi	NE	Omnivore	FRE; BEP
355	Rhinogobius giurinus	LC	Carnivore	MAR; FRE; BRA; DEM; AMP
	Sicydium			
356	Sicydium brevifile	LC	Omnivore	FRE; DEM; AMP
	Sicyopterus			
357	Sicyopterus japonicus	NE	Detrivore	MAR; FRE; BRA; DEM; AMP
358	Sicyopterus lagocephalus	LC	Herbivore	MAR; FRE; BRA; DEM; AMP
359	Sicyopterus pugnans	LC	Omnivore	FRE; DEM
360	Sicyopus zosterophorus	LC	Omnivore	MAR; FRE; BRA; BEP; AMP
	Stiphodon			
361	Stiphodon elegans	LC	Detrivore	MAR; FRE; BRA; DEM
	Synechogobius			
362	Synechogobius ommaturus	NE	Omnivore	FRE; BRA; DEM
	Taenioides			
363	Taenioides anguillaris	LC	Omnivore	MAR; FRE; BRA; DEM
364	Taenioides cirratus	DD	Carnivore	MAR; FRE; BRA; DEM; AMP
	Tridentiger			
365	Tridentiger barbatus	NE	Omnivore	BRA; DEM
366	Tridentiger bifasciatus	LC	Omnivore	MAR; FRE; BRA; DEM; AMP
367	Tridentiger obscurus	NE	Carnivore	MAR; FRE; BRA; DEM; AMP
368	Tridentiger trigonocephalus	LC	Omnivore	MAR; FRE; BRA; DEM
	Trypauchen			

No.	Class, Order, Family, Genus, Species	IUCN	Feeding habits	Habitat
369	Trypauchen vagina	LC	Carnivore	MAR; BRA; DEM
	Wuhanlinigobius			
370	Wuhanlinigobius polylepis	NE	Omnivore	MAR; BRA; DEM
	Yongeichthys			
371	Yongeichthys criniger	NE	Carnivore	MAR; BRA; DEM
	Haemulidae			
	Diagramma			
372	Diagramma pictum	NE	Omnivore	MAR; RFA
	Hapalogenys			
373	Hapalogenys analis	NE	Carnivore	MAR; BEP
374	Hapalogenys nigripinnis	NE	Carnivore	MAR; BRA; BEP
	Parapristipoma			
375	Parapristipoma trilineatum	NE	Carnivore	MAR; BEP; OD
	Plectorhinchus			
376	Plectorhinchus cinctus	NE	Carnivore	MAR; RFA
	Pomadasys			
377	Pomadasys argenteus	LC	Carnivore	MAR; FRE; BRA; DEM
378	Pomadasys kaakan	NE	Carnivore	MAR; BRA; RFA
379	Pomadasys maculatus	LC	Carnivore	MAR; BRA; RFA; AMP
	Kyphosidae			
	Kyphosus			
380	Kyphosus vaigiensis	LC	Detrivore	MAR; RFA
	Labridae			
	Choerodon			
381	Choerodon schoenleinii	NT	Carnivore	MAR; RFA
	Halichoeres			
382	Halichoeres argus	LC	Omnivore	MAR; RFA
383	Halichoeres nigrescens	LC	Omnivore	MAR; RFA
	Stethojulis			
384	Stethojulis strigiventer	LC	Carnivore	MAR; RFA
385	Stethojulis terina	LC	Carnivore	MAR; RFA
	Lactariidae			
	Lactarius			
386	Lactarius lactarius	NE	Carnivore	MAR; BRA; PE
	Latidae			
	Lates			
387	Lates calcarifer*	LC	Carnivore	MAR; FRE; BRA; DEM
	Psammoperca			
388	Psammoperca waigiensis	NE	Carnivore	MAR; BRA; RFA
	Leiognathidae			
	Deveximentum			
389	Deveximentum insidiator	NE	Carnivore	MAR; BRA; DEM
	Equulites			
390	Equulites elongatus	NE	Omnivore	MAR; DEM
391	Equulites lineolatus	NE	Carnivore	MAR; DEM
392	Equulites rivulatus	NE	Carnivore	MAR; DEM
	Eubleekeria			

No.	Class, Order, Family, Genus, Species	IUCN	Feeding habits	Habitat
393	Eubleekeria splendens	LC	Herbivore	MAR; BRA; DEM
	Gazza			
394	Gazza achlamys	LC	Carnivore	MAR; BRA; RFA
395	Gazza minuta	LC	Carnivore	MAR; BRA; DEM
	Karalla			
396	Karalla daura	NE	Carnivore	MAR; DEM
397	Karalla dussumieri	NE	Carnivore	MAR; BRA; DEM
	Leiognathus			
398	Leiognathus berbis	NE	Carnivore	MAR; BRA; DEM
399	Leiognathus brevirostris	NE	Carnivore	MAR; BRA; DEM
400	Leiognathus equulus	LC	Carnivore	MAR; FRE; BRA; DEM; AMP
401	Leiognathus ruconius	NE	Carnivore	MAR; FRE; BRA; DEM
	Nuchequula			
402	Nuchequula nuchalis	NE	Carnivore	MAR; BRA
	Photopectoralis			
403	Photopectoralis bindus	NE	Herbivore	MAR; BRA; DEM
	Lethrinidae			
	Lethrinus			
404	Lethrinus haematopterus	NE	Omnivore	MAR; RFA
405	Lethrinus harak	LC	Carnivore	MAR; BRA; RFA
406	Lethrinus lentjan	LC	Carnivore	MAR; BRA; RFA
	Lutjanidae			
	Lutjanus			
407	Lutjanus argentimaculatus	LC	Carnivore	MAR; FRE; BRA; RFA
408	Lutjanus fulviflamma	LC	Carnivore	MAR; BRA; RFA
409	Lutjanus fulvus	LC	Carnivore	MAR; FRE; BRA; RFA
410	Lutjanus johnii	LC	Carnivore	MAR; BRA; RFA
411	Lutjanus kasmira	LC	Carnivore	MAR; RFA
412	Lutjanus ophuysenii	NE	Omnivore	MAR; DEM
413	Lutjanus quinquelineatus	LC	Carnivore	MAR; RFA
414	Lutjanus russellii	LC	Carnivore	MAR; BRA; RFA
	Pristipomoides			
415	Pristipomoides filamentosus	LC	Carnivore	MAR; BEP
	Malacanthidae			
	Branchiostegus			
416	Branchiostegus japonicus	LC	Carnivore	MAR; DEM
	Malacanthus			
417	Malacanthus latovittatus	NE	Carnivore	MAR; RFA
	Menidae			
	Mene			
418	Mene maculata	NE	Carnivore	MAR; BRA; RFA
	Monodactylidae			
	Monodactylus			
419	Monodactylus argenteus*	LC	Carnivore	MAR; FRE; BRA; PE
	Moronidae			
	Lateolabrax			

No.	Class, Order, Family, Genus, Species	IUCN	Feeding habits	Habitat
420	Lateolabrax japonicus	NE	Carnivore	MAR; FRE; BRA; RFA
	Mullidae			
	Parupeneus			
421	Parupeneus ciliatus	LC	Carnivore	MAR; RFA
422	Parupeneus forsskali	LC	Carnivore	MAR; BRA; RFA
423	Parupeneus multifasciatus	LC	Carnivore	MAR; RFA
	Pseudupeneus			
424	Pseudupeneus prayensis	VU	Carnivore	MAR; DEM
	Upeneus			
425	Upeneus japonicus	NE	Carnivore	MAR; RFA
426	Upeneus quadrilineatus	NE	Omnivore	MAR; DEM
427	Upeneus sulphureus	LC	Carnivore	MAR; BRA; DEM; OD
428	Upeneus tragula	LC	Carnivore	MAR; BRA; RFA; OD
	Nemipteridae			
	Nemipterus			
429	Nemipterus peronii	LC	Carnivore	MAR; BRA; DEM
430	Nemipterus virgatus	VU	Carnivore	MAR; DEM
	Parascolopsis			
431	Parascolopsis inermis	LC	Carnivore	MAR; DEM
	Pentapodus			
432	Pentapodus setosus	NE	Carnivore	MAR; RFA
	Scolopsis			
433	Scolopsis monogramma	LC	Carnivore	MAR; RFA
434	Scolopsis taeniopterus	NE	Carnivore	MAR; DEM
435	Scolopsis vosmeri	LC	Carnivore	MAR; RFA
	Osphronemidae			
	Macropodus			
436	Macropodus opercularis	LC	Carnivore	FRE; BRA; PEL
	Pempheridae			
	Pempheris			
437	Pempheris oualensis	NE	Carnivore	MAR; RFA
	Periophthalmidae			
	Boleophthalmus			
438	Boleophthalmus pectinirostris	NE	Detrivore	MAR; FRE; BRA; DEM
	Scartelaos			
439	Scartelaos gigas	NE	Omnivore	MAR; DEM
440	Scartelaos histophorus	LC	Omnivore	MAR; BRA; DEM
	Pinguipedidae			
	Parapercis			
441	Parapercis ommatura	NE	Omnivore	MAR; DEM
442	Parapercis sexfasciata	NE	Omnivore	MAR; DEM
	Pomacentridae			
	Abudefduf			
443	Abudefduf sordidus	LC	Herbivore	MAR; BRA; RFA
444	Abudefduf vaigiensis	LC	Herbivore	MAR; RFA; OD
	Neoglyphidodon			
445	Neoglyphidodon melas	NE	Carnivore	MAR; RFA

No.	Class, Order, Family, Genus, Species	IUCN	Feeding habits	Habitat
	Stegastes			
446	Stegastes fasciolatus	NE	Detrivore	MAR; RFA
	Priacanthidae			
	Priacanthus			
447	Priacanthus macracanthus	LC	Carnivore	MAR; RFA
	Ptereleotridae			
	Parioglossus			
448	Parioglossus dotui	NE	Omnivore	MAR; BRA; BEP
	Rachycentridae			
	Rachycentron			
449	Rachycentron canadum	LC	Carnivore	MAR; BRA; RFA
	Rhyacichthyidae			
	Rhyacichthys			
450	Rhyacichthys aspro	DD	Omnivore	MAR; FRE; BRA; DEM; AMP
	Scaridae			
	Leptoscarus			
451	Leptoscarus vaigiensis	LC	Detrivore	MAR; RFA
	Scarus			
452	Scarus ghobban	LC	Detrivore	MAR; BRA; RFA
	Scatophagidae			
	Scatophagus			
453	Scatophagus argus	LC	Herbivore	MAR; FRE; BRA; RFA
	Selenotoca			
454	Selenotoca multifasciata	LC	Omnivore	MAR; FRE; BRA; BEP; AMP
	Sciaenidae			
	Chrysochir			
455	Chrysochir aureus	LC	Carnivore	MAR; BRA; BEP
	Collichthys			
456	Collichthys lucidus	LC	Carnivore	MAR; DEM; OD
	Dendrophysa			
457	Dendrophysa russelii	LC	Omnivore	MAR; FRE; BRA; DEM; AMP
	Johnius			
458	Johnius belangerii	LC	Carnivore	MAR; BRA; DEM; AMP
459	Johnius distinctus	LC	Omnivore	MAR; BEP
460	Johnius fasciatus	NE	Omnivore	FRE; BRA; DEM
	Larimichthys			
461	Larimichthys crocea	CR	Carnivore	MAR; BRA; BEP; OD
462	Larimichthys polyactis	LC	Carnivore	MAR; BEP; OD
	Macrospinosa			
463	Macrospinosa cuja	DD	Omnivore	MAR; FRE; BRA
	Nibea			
464	Nibea albiflora	LC	Carnivore	MAR; BEP
465	Nibea coibor	DD	Omnivore	MAR; BRA; DEM
	Otolithes			
466	Otolithes ruber	LC	Carnivore	MAR; BRA; BEP; AMP
	Pennahia			

No.	Class, Order, Family, Genus, Species	IUCN	Feeding habits	Habitat
467	Pennahia anea	LC	Carnivore	MAR; DEM
468	Pennahia argentata	LC	Carnivore	MAR; BEP; OD
469	Pennahia macrocephalus	LC	Carnivore	MAR; DEM
470	Pennahia pawak	LC	Carnivore	MAR; BEP
	Protonibea			
471	Protonibea diacanthus	NT	Carnivore	MAR; BRA; DEM
	Sciaenops			
472	Sciaenops ocellatus*	LC	Carnivore	MAR; BRA; DEM; OD
	Scombridae			
	Euthynnus			
473	Euthynnus affinis	LC	Carnivore	MAR; PE; OD
	Rastrelliger			
474	Rastrelliger kanagurta	DD	Carnivore	MAR; PE; OD
	Scomber			
475	Scomber australasicus	LC	Carnivore	MAR; PE; OD
476	Scomber japonicus	LC	Carnivore	MAR; PE; OD
	Scomberomorus			
477	Scomberomorus niphonius	DD	Carnivore	MAR; PE; OD
	Serranidae			
	Epinephelus			
478	Epinephelus akaara	EN	Carnivore	MAR; RFA
479	Epinephelus bleekeri	DD	Omnivore	MAR; DEM
480	Epinephelus coioides	LC	Carnivore	MAR; BRA; RFA
481	Epinephelus fasciatomaculosus	LC	Carnivore	MAR; RFA
482	Epinephelus lanceolatus	DD	Carnivore	MAR; BRA; RFA
483	Epinephelus malabaricus	LC	Carnivore	MAR; BRA; RFA; AMP
484	Epinephelus quoyanus	LC	Carnivore	MAR; RFA
485	Epinephelus sexfasciatus	LC	Carnivore	MAR; RFA
486	Epinephelus trimaculatus	LC	Carnivore	MAR; RFA
	Grammistes			
487	Grammistes sexlineatus	LC	Carnivore	MAR; RFA
	Plectranthias			
488	Plectranthias japonicus	NE	Carnivore	MAR; DEM
	Siganidae			
	Siganus			
489	Siganus argenteus	LC	Detrivore	MAR; RFA
490	Siganus canaliculatus	LC	Detrivore	MAR; BRA; RFA
491	Siganus fuscescens	LC	Detrivore	MAR; BRA; RFA
492	Siganus guttatus	LC	Detrivore	MAR; BRA; RFA
	Sillaginidae			
	Sillago			
493	Sillago aeolus	NE	Omnivore	MAR; DEM
494	Sillago asiatica	NE	Omnivore	MAR; DEM
495	Sillago japonica	LC	Carnivore	MAR; DEM
496	Sillago maculata	NE	Carnivore	MAR; BRA; DEM
497	Sillago sihama	LC	Carnivore	MAR; BRA; RFA; AMP
	Sparidae			

No.	Class, Order, Family, Genus, Species	IUCN	Feeding habits	Habitat
	Acanthopagrus			
498	Acanthopagrus berda	LC	Carnivore	MAR; FRE; BRA; DEM
499	Acanthopagrus chinshira	NT	Omnivore	MAR; PE
500	Acanthopagrus latus	DD	Carnivore	MAR; FRE; BRA; DEM
501	Acanthopagrus schlegelii	LC	Carnivore	MAR; BRA; DEM
	Dentex			
502	Dentex hypselosomus	LC	Carnivore	MAR; DEM
	Pagrus			
503	Pagrus major	LC	Carnivore	MAR; DEM
	Parargyrops			
504	Parargyrops edita	NE	Omnivore	
	Rhabdosargus			
505	Rhabdosargus sarba	LC	Carnivore	MAR; BRA; RFA
	Sparus			
506	Sparus aurata*	LC	Carnivore	MAR; BRA; DEM
	Sphyraenidae			
	Sphyraena			
507	Sphyraena barracuda	LC	Carnivore	MAR; BRA; RFA
508	Sphyraena flavicauda	NE	Carnivore	MAR; RFA
509	Sphyraena jello	NE	Carnivore	MAR; BRA; RFA
510	Sphyraena qenie	NE	Carnivore	MAR; RFA
	Stromateidae			
	Pampus			
511	Pampus argenteus*	NE	Carnivore	MAR; BEP
512	Pampus chinensis	NE	Carnivore	MAR; BRA
513	Pampus cinereus	NE	Carnivore	MAR; BEP
514	Pampus nozawae	NE	Carnivore	
	Terapontidae			
	Helotes			
515	Helotes sexlineatus	LC	Detrivore	MAR; BRA; BEP
	Pelates			
516	Pelates quadrilineatus	NE	Carnivore	MAR; BRA; RFA
	Rhynchopelates			
517	Rhynchopelates oxyrhynchus	NE	Carnivore	MAR; FRE; BRA; BEP
	Terapon			
518	Terapon jarbua	LC	Carnivore	MAR; FRE; BRA; DEM
519	Terapon theraps	LC	Carnivore	MAR; FRE; BRA; RFA
	Trichiuridae			
	Eupleurogrammus			
520	Eupleurogrammus muticus	NE	Carnivore	MAR; BRA; BEP
	Lepturacanthus			
521	Lepturacanthus savala	NE	Carnivore	MAR; BRA; BEP
	Trichiurus			
522	Trichiurus lepturus	LC	Carnivore	MAR; BRA; BEP
	Pleuronectiformes			
	Bothidae			

No.	Class, Order, Family, Genus, Species	IUCN	Feeding habits	Habitat
	Arnoglossus			
523	Arnoglossus tenuis	LC	Carnivore	MAR; DEM
	Psettina			
524	Psettina iijimae	LC	Carnivore	MAR; DEM
	Cynoglossidae			
	Cynoglossus			
525	Cynoglossus abbreviatus	NE	Carnivore	MAR; DEM
526	Cynoglossus arel	DD	Carnivore	MAR; BRA; DEM
527	Cynoglossus bilineatus	NE	Carnivore	MAR; BRA; DEM
528	Cynoglossus itinus	NE	Omnivore	MAR; DEM
529	Cynoglossus joyneri	NE	Carnivore	MAR; DEM
530	Cynoglossus puncticeps	LC	Carnivore	MAR; FRE; BRA; DEM
531	Cynoglossus robustus	NE	Omnivore	MAR; DEM
532	Cynoglossus semilaevis	NE	Carnivore	MAR; FRE; BRA; DEM
533	Cynoglossus sinicus	NE	Omnivore	FRE; BRA; DEM
534	Cynoglossus trigrammus	LC	Omnivore	FRE; BRA; DEM
	Paraplagusia			
535	Paraplagusia bilineata	NE	Omnivore	MAR; BRA; DEM
536	Paraplagusia blochii	NE	Omnivore	MAR; BRA; DEM
	Paralichthyidae			
	Paralichthys			
537	Paralichthys olivaceus	NE	Carnivore	MAR; DEM
	Pseudorhombus			
538	Pseudorhombus arsius	NE	Carnivore	MAR; BRA; DEM
539	Pseudorhombus cinnamoneus	LC	Carnivore	MAR; DEM
540	Pseudorhombus dupliciocellatus	NE	Carnivore	MAR; DEM
541	Pseudorhombus elevatus	NE	Carnivore	MAR; DEM
542	Pseudorhombus levisquamis	LC	Omnivore	MAR; DEM
543	Pseudorhombus malayanus	NE	Carnivore	MAR; DEM
544	Pseudorhombus oligodon	LC	Carnivore	MAR; DEM
	Pleuronectidae			
	Eopsetta			
545	Eopsetta grigorjewi	NE	Omnivore	MAR; DEM
	Pleuronichthys			
546	Pleuronichthys cornutus	NE	Omnivore	MAR; DEM
	Samaris			
547	Samaris cristatus	LC	Carnivore	MAR; BRA; DEM
	Psettodidae			
	Psettodes			
548	Psettodes erumei	DD	Carnivore	MAR; DEM
	Samaridae			
	Plagiopsetta			
549	Plagiopsetta glossa	LC	Carnivore	MAR; DEM
	Soleidae			
	Aseraggodes			
550	Aseraggodes orientalis	DD	Omnivore	MAR; RFA
	Brachirus			

No.	Class, Order, Family, Genus, Species	IUCN	Feeding habits	Habitat
551	Brachirus orientalis	LC	Carnivore	MAR; FRE; BRA; DEM
552	Brachirus pan	LC	Omnivore	MAR; FRE; BRA; DEM
	Pardachirus			
553	Pardachirus pavoninus	LC	Carnivore	MAR; RFA
	Solea			
554	Solea ovata	LC	Carnivore	MAR; DEM
	Zebrias			
555	Zebrias quagga	LC	Carnivore	MAR; DEM
556	Zebrias zebra	NE	Carnivore	MAR; BRA; RFA
	Scorpaeniformes			
	Aploactinidae			
	Acanthosphex			
557	Acanthosphex leurynnis	LC	Omnivore	MAR; DEM
	Hypodytes			
558	Hypodytes indicus	NE	Omnivore	
	Bembridae			
	Bembras			
559	Bembras japonica	NE	Omnivore	MAR; DEM
	Platycephalidae			
	Cociella			
560	Cociella crocodila	LC	Carnivore	MAR; BRA; RFA
	Grammoplites			
561	Grammoplites scaber	NE	Omnivore	MAR; BRA; DEM; AMP
	Inegocia			
562	Inegocia japonica	LC	Carnivore	MAR; DEM
	Onigocia			
563	Onigocia macrolepis	LC	Carnivore	MAR; DEM
	Platycephalus			
564	Platycephalus indicus	DD	Carnivore	MAR; BRA; RFA; OD
	Scorpaenidae			
	Inimicus			
565	Inimicus japonicus	NE	Carnivore	MAR; DEM
	Minous			
566	Minous monodactylus	LC	Omnivore	MAR; DEM
567	Minous trachycephalus	NE	Omnivore	MAR; DEM
	Paracentropogon			
568	Paracentropogon longispinis	LC	Omnivore	MAR; DEM
	Scorpaenopsis			
569	Scorpaenopsis neglecta	LC	Omnivore	MAR; DEM
	Sebastiscus			
570	Sebastiscus albofasciatus	NE	Omnivore	MAR; DEM
571	Sebastiscus marmoratus	NE	Omnivore	MAR; DEM; OD
	Tetraroge			
572	Tetraroge barbata	NE	Carnivore	MAR; FRE; BRA; RFA
	Trachicephalus			
573	Trachicephalus uranoscopus	NE	Omnivore	MAR; BRA; DEM

No.	Class, Order, Family, Genus, Species	IUCN	Feeding habits	Habitat
	Vespicula			
574	Vespicula trachinoides	NE	Omnivore	MAR; BRA; DEM
	Triglidae			
	Chelidonichthys			
575	Chelidonichthys kumu	LC	Carnivore	MAR; BRA; DEM
	Lepidotrigla			
576	Lepidotrigla oglina	NE	Omnivore	MAR; BAD
	Siluriformes			
	Ariidae			
	Arius			
577	Arius arius	LC	Carnivore	MAR; BRA; DEM; AMP
578	Arius maculatus	NE	Carnivore	MAR; FRE; BRA; DEM; POT
	Netuma			
579	Netuma thalassina	NE	Carnivore	MAR; FRE; BRA; DEM; AMP
	Plicofollis			
580	Plicofollis nella	NE	Omnivore	MAR; BRA; DEM
	Bagridae			
	Tachysurus			
581	Tachysurus sinensis	NE	Carnivore	FRE; BRA; DEM
	Clariidae			
	Clarias			
582	Clarias fuscus	LC	Carnivore	FRE; DEM
	Loricariidae			
	Hypostomus			
583	Hypostomus Plecostomus*	NE	Herbivore	FRE; DEM
	Plotosidae			
	Plotosus			
584	Plotosus lineatus	NE	Carnivore	MAR; BRA; RFA; AMP
	Siluridae			
	Silurus			
585	Silurus asotus	LC	Carnivore	FRE; DEM
	Stomiiformes			
	Phosichthyidae			
	Vinciguerria			
586	Vinciguerria nimbaria	LC	Carnivore	MAR; BAP
	Synbranchiformes			
	Synbranchidae			
	Monopterus			
587	Monopterus albus	LC	Carnivore	FRE; BRA; DEM
	Tetraodontiformes			
	Monacanthidae			
	Aluterus			
588	Aluterus monoceros	LC	Carnivore	MAR; RFA
	Monacanthus			
589	Monacanthus chinensis	LC	Herbivore	MAR; RFA
	Paramonacanthus			
590	Paramonacanthus sulcatus	LC	Omnivore	MAR; BEP

No.	Class, Order, Family, Genus, Species	IUCN	Feeding habits	Habitat
	Pseudomonacanthus			
591	Pseudomonacanthus peroni	LC	Omnivore	MAR; RFA
	Thamnaconus			
592	Thamnaconus septentrionalis	LC	Omnivore	MAR; DEM
593	Thamnaconus tessellatus	LC	Omnivore	MAR; BAD
	Ostraciontidae			
	Ostracion			
594	Ostracion immaculatus	NE	Omnivore	MAR; DEM
	Tetraodontidae			
	Amblyrhynchotes			
595	Amblyrhynchotes honckenii	LC	Omnivore	
	Arothron			
596	Arothron hispidus	LC	Herbivore	MAR; BRA; RFA
597	Arothron immaculatus	LC	Herbivore	MAR; BRA; RFA
	Chelonodon			
598	Chelonodon patoca	LC	Carnivore	MAR; FRE; BRA; RFA; ANA
	Lagocephalus			
599	Lagocephalus inermis	LC	Omnivore	MAR; DEM
600	Lagocephalus lunaris	LC	Omnivore	MAR; BRA; DEM; OD
601	Lagocephalus spadiceus	LC	Omnivore	MAR; BRA; DEM; OD
	Takifugu			
602	Takifugu alboplumbeus	LC	Omnivore	MAR; DEM
603	Takifugu basilevskianus	DD	Omnivore	MAR; RFA
604	Takifugu bimaculatus	LC	Omnivore	MAR; DEM
605	Takifugu niphobles	LC	Carnivore	MAR; DEM
606	Takifugu oblongus	LC	Carnivore	MAR; BRA; DEM
607	Takifugu ocellatus	NT	Omnivore	MAR; FRE; BRA; DEM; ANA
608	Takifugu porphyreus	LC	Omnivore	MAR; DEM
609	Takifugu xanthopterus	LC	Carnivore	MAR; DEM
	Triacanthidae			
	Triacanthus			
610	Triacanthus biaculeatus	NE	Carnivore	MAR; BRA; DEM
611	Triacanthus nieuhofii	NE	Carnivore	MAR; DEM

Note: The Habitats are indicated as: Brackish (BRA); reef-associated (RFA); oceanodromous (OD); amphidromous (AMP); anadromous (ANA); pelagic-neritic (PE); bathypelagic (BAP); benthopelagic (BEP); pelagic-oceanic (PELO); pelagic (PEL); demersal (DEM); bathydemersal (BAD); Marine (MAR); freshwater (FRE); potamodromous (POT). The IUCN status are indicated as: Critically Endangered (CR); Endangered (EN); Vulnerable (VU); Near Threatened (NT); Least Concern (LC); Data Deficient (DD); Not Evaluated (NE). *: Non-native species.
